# Supplementary material for: Frequency of MTB and rifampicin resistance MTB using Xpert-MTB/RIF assay among adult presumptive tuberculosis patients in Tigray, Northern Ethiopia: A cross sectional study
Source: PLoS One. 2020 Nov 4;15(11):e0240361. doi: 10.1371/journal.pone.0240361 (PMC7641410; doi:10.1371/journal.pone.0240361)
Supplement: Attachment — Submitted filename: Rif comment.docx [file pone.0240361.s001.docx]

Despite you try TO deal with the current national and global issue, the drug resistance TB, but your manuscript is not well written. I presented some of my comments, but many still left,

General comments

- Your writing is not consistent terms. E,g since xpert detect only *mycobacterium* *tuberculosis* it should be written as “RR_MTB,” but you randomly wrote as RR-TB, RR-MTB. It should be consistent though out the manuscript
- There may vocabulary / grammar errors. Incases MTP instead MTB
- You use your non standard terminologies: RR-TB positive AND RR-TB negative (table 1). RR_MTB DETECTED OR RR_MTB NOT DETECTED
- The texts/ paragraph with our references e.g “….Most studies in Ethiopia used the conventional culture and sensitivity methods not the automated Xpert MTB/RIF assay…” (Introduction). There are also reliable references. E.g there reference for population size of 6,960,000 of the region is the study from south Africa “Group Accuracy of the Xpert MTB / RIF test for the diagnosis of pulmonary tuberculosis in children admitted to hospital in Cape Town , South Africa : a descriptive study” . All the reference should be checked and reliable

**Abstract**

1. Part of the conclusion is not drawn from your data. In cases you try to highlights the need of a coordinated work in health education despite your data not support it

**Methodology**

1. The study setting is confusing : your describe that there 12 general hospitals, 22 primary hospitals,…… in the region” . You collect data from the five general hospitals and one compressive hospital. On the pagra 3 you’re said “There are two general hospitals and one primary hospital which are not included in the study”.
2. Inclusion criteria;-despite you used secondary data (log book) but you exclude children and sample other than the sputum. Why ?
3. **Laboratory Processing: it says “**Samples were collected before the patients started anti-TB treatment” but on your result there are patents with failure and lost who already on treatment but sample collected for the indication probably as per national protocol
4. **HIV testing:** you describe the algorism which could potential be modified as per the change in technology without reference**.**
5. **Quality control: “…** the researchers checked and confirmed that the Gene Xpert MTB/RIF assay was done using standard operating procedures”. How it possible for the secondary data? You can obtain SOP but how you know whether they followed or not. Such unnecessary should be modified

**Result**

1. **Socio-demographic, Clinical characteristics and TB results**

- Using **median** instead of the **mean** is more appropriate for your study as the age range 18-112, which seems having extreme age like 112.
- The writing style not consistent. E.g (17,471; 57.7 %), 28,996 (95.7 %), 7.9 %

2. **Associated Risk Factors of MTB or RR_ MTB Infections**

- Interpretation of the statistical finding **wrong**. E.G for AOR of 0.86, your describe as “…females were 86 % times less likely to be infected by TB compared to males”. Rather is should be	… female has 26% less likely infected by TB compared to males”. Many other examples found in your result section while you interpreted the associated Risk Factors of for MTB	AND RR –TB. This misleading interpretation also reflected in your discussion part.
